# The Efficacy of Gelam Honey Dressing towards Excisional Wound Healing

**DOI:** 10.1155/2012/805932

**Published:** 2012-03-28

**Authors:** Mui Koon Tan, Durriyyah Sharifah Hasan Adli, Mohd Amzari Tumiran, Mahmood Ameen Abdulla, Kamaruddin Mohd Yusoff

**Affiliations:** ^1^Division of Biohealth Science, Institute of Biological Sciences, Faculty of Science, University of Malaya, 50603 Kuala Lumpur, Malaysia; ^2^Institute of Graduate Studies, University of Malaya, 50603 Kuala Lumpur, Malaysia; ^3^Department of Molecular Medicine, Faculty of Medicine, University of Malaya, 50603 Kuala Lumpur, Malaysia

## Abstract

Honey is one of the oldest substances used in wound management. Efficacy of Gelam honey in wound healing was evaluated in this paper. *Sprague-Dawley* rats were randomly divided into four groups of 24 rats each (untreated group, saline group, Intrasite Gel group, and Gelam honey group) with 2 cm by 2 cm full thickness, excisional wound created on neck area. Wounds were dressed topically according to groups. Rats were sacrificed on days 1, 5, 10, and 15 of treatments. Wounds were then processed for macroscopic and histological observations. Gelam-honey-dressed wounds healed earlier (day 13) than untreated and saline treated groups, as did wounds treated with Intrasite Gel. Honey-treated wounds exhibited less scab and only thin scar formations. Histological features demonstrated positive effects of Gelam honey on the wounds. This paper showed that Gelam honey dressing on excisional wound accelerated the process of wound healing.

## 1. Introduction

Wound healing is a complex biological cascade of cellular and biochemical events comprised of three phases: inflammation, proliferation, and maturation [1, 2]. Despite recent advances in health care, inadequate wound management and development of secondary infections leading to increased morbidity is still a major public health problem in the world, especially in developing countries. Hence, wound management still remains an important focus of researches [2, 3]. Recently, the interest of using alternative therapies and natural remedies in wound management has rapidly increased. Alternative methods have great potential to improve wound healing for global population as they reduce financial burden of modern treatments [2, 4]. One of the natural products of interest in this regard is honey which had attracted the attention of many researchers [2, 5]. 

Honey has been used for its medicinal properties in many cultures since ancient times [5, 6]. It is one of the oldest and most enduring-substance used in wound management. The effectiveness of honey in wound healing is attributed to several factors such as its antibacterial activity, antioxidant activity, stimulatory effects, and anti-inflammatory effects. A rapid increase of interest in the use of honey as wound dressing among researchers and modern practitioners [7] includes case studies and clinical trials reporting the effectiveness of honey in the treatment of different types of wounds, with some showing effectiveness against bacterial strains resistant to synthetic antibiotics [2]. Scientists found that different types of honey differ substantially in their activities, partly depending on their source.

In Malaysia, a variety of honey is locally produced. Unfortunately, there is still little evidence to support the potential of Malaysian honey on wound healing. Thus, this present study was designed to evaluate the efficacy of a selected Malaysian honey towards excisional wound healing.

## 2. Materials and Methods

### 2.1. Honey Sample

Gelam honey is a local monofloral honey produced by *Apis mellifera* bees from the flora source of Gelam (*Melaleuca spp.*) tree. This honey was chosen as treatment in this study. This honey was obtained from an Apiary at Malaysian Agriculture Department. The honey was obtained by the normal procedure of centrifuging the cut comb in a stainless steel container and filtered once by using a fine muslin cloth. The honey used was sterilized by gamma-irradiation (25 kGy) and kept at laboratory temperature (20°C), away from direct sunlight in aluminum foil covered glass bottle.

### 2.2. Intrasite Gel

This wound dressing is a clear amorphous hydrogel containing modified carboxymethyl cellulose polymer, propylene glycol, and water. It provides a moist environment which is suitable for wound healing and promotes natural debridement by rehydrating necrotic tissue. Intrasite Gel (Smith & Nephew) was obtained from the pharmacy of University of Malaya Medical Centre.

### 2.3. Experimental Animal

Adult male *Sprague-Dawley* rats weighing between 180 and 250 g were used in this study. These rats were obtained from the Animal House, Faculty of Medicine, University of Malaya. They were caged separately and alone in individual wire-bottom cages to prevent them from fighting and biting each other's wound. The rats were kept under standard 12/12 light/dark cycle, fed with standard pellet diet and tap water *ad libitum*. No changes were observed in their diet intake. The cages and the air-ventilated room were cleaned daily to prevent the unwanted infection to the wound.

### 2.4. Experimental Wound Model and Wounding Procedure

**Figure 1:**
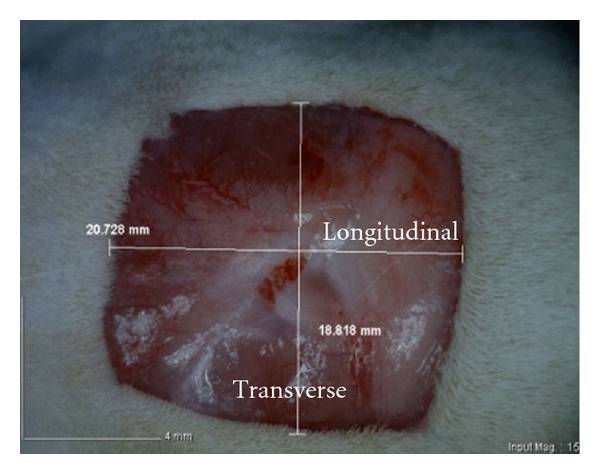
2 cm by 2 cm excision skin wound on day 0, before application of treatments.

Uninfected, 4 cm^2^ full thickness, excisional wounds were used in this study to represent the acute wound healing (Figure 1).

This experimental protocol was adapted and modified from Aljady et al. [2] and Tomlinson and Ferguson [8]. The protocol was approved by the Ethics Committee for Animal experimentation of Faculty of Medicine, University of Malaya (reference no.: ethics no. PM/27/01/2010/KMY(R)). 

The wounding procedure was carried out under general anesthesia by using Ketamine 50 mg/kg and Xylazine 5 mg/kg in mix in same syringe-injected intramuscularly, producing 30 minutes of anesthesia. Dorsum area of the rats was shaved by electrical hair clipper and swabbed with 70% alcohol and 1 mL of Lignocaine HCl (2%, 100 mL/5 mL) was injected subcutaneously for local anesthesia. A square wound (2 cm by 2 cm) was marked by using wound mold on the posterior neck area between the shoulders and excised carefully by using a pair of surgical scissors aseptically. The wound mold did not contact the wound, since it was only used in marking the border of the wound before the surgery. The location of the wound area was chosen so as to prevent unwanted wound caused by stretching and biting from the rat, itself. The mold, scissors, and forceps were cleansed with 95% alcohol after each use. All wounds were of full thickness extending vertically down to the subcutaneous tissue.

### 2.5. Grouping, Mode of Treatment, and Sampling

Treatments started 24 hours after wound creation, the wounds were air exposed, and dressing was applied topically once a day. The rats were randomly divided into four experimental groups with 24 animals per group. Untreated group was left without any treatment to serve as the untreated control group. Saline group was treated topically with normal saline (negative control), Intrasite group was treated with Intrasite Gel (positive control), while Gelam group (experimental group) was treated with Gelam honey.

Six rats from each group were sacrificed at days 1, 5, 10, and 15 of treatments. Entire wound tissue area was removed carefully from each rat and fixed immediately for histological process. All samples were properly labeled with unique numbers before storage and the measurements were done at random to overcome experimental bias.

### 2.6. Assessment of Wound Healing

Photograph of each wound was taken for analytical purposes, and macroscopic evaluation (e.g., wound appearance) was recorded. All wounds were assessed clinically according to the scoring system modified from the clinical judgment by Bates-Jensen [9] and Khoo et al. [10].

On days 1, 5, 10, and 15 of treatments, relevant groups had their wounds measured before tissues were excised. Animals were anesthetized during the procedures. Margin of the wounds was traced on a transparency paper by a fine tip permanent marker for the evaluation of the rate of wound contraction. Contraction of both longitudinal (length) and transverse (width) measurements of wounds was recorded. Wound contraction was calculated as described by Aljady et al. [2]. As for the wound area, it was measured by square counting procedure according to Aljady et al. [2], modified from Schubert [11] and Richard et al. [12]. The number of squares (0.04 cm^2^) that appeared completely (*N*_*c*_) and partially (*N*_*p*_) inside the tracing was counted, and the area size was determined using the following formula: *A*_*c*+*p*_ = (*N*_*c*_ + 0.4 × *N*_*p*_) × 0.04.

### 2.7. Histological Evaluation of Healed Wounds

Excised tissue was fixed in 10% formalin before histologically processed. Sections were made at the thickness of 5 *μ*m for Hematoxylin and Eosin (H&E) staining. Assessment for histological features was made. The histological slides were observed under light microscopy (Olympus BX51) and image captured using Olympus analySIS LifeScience Research Imaging System.

### 2.8. Statistical Analysis

All values were reported as Mean ± Standard Error Mean (S.E.M.). The statistical differences among groups were assessed using one-way ANOVA (analysis of variance). A value of *P* < 0.05 was considered significant. Statistical analysis was performed using SPSS statistical software for Windows (SPSS Inc., Chicago, IL, USA).

## 3. Results

### 3.1. Wound Healing Time

The effects of various treatments on duration of wound healing process were shown in Table 1. Use of Intrasite Gel and Gelam honey showed significant (*P* < 0.05) decrease in wound healing compared to the no treatment and saline treatment. There was no significant difference in the duration of wound healing between the groups treated with Intrasite Gel and Gelam honey: both healed by about 13 days. Untreated wound, however, needed around 16 days to heal: about three days longer than the wound healing time needed under Intrasite Gel and Gelam honey treatments.

### 3.2. Macroscopic and Histological Evaluation

**Figure 2:**
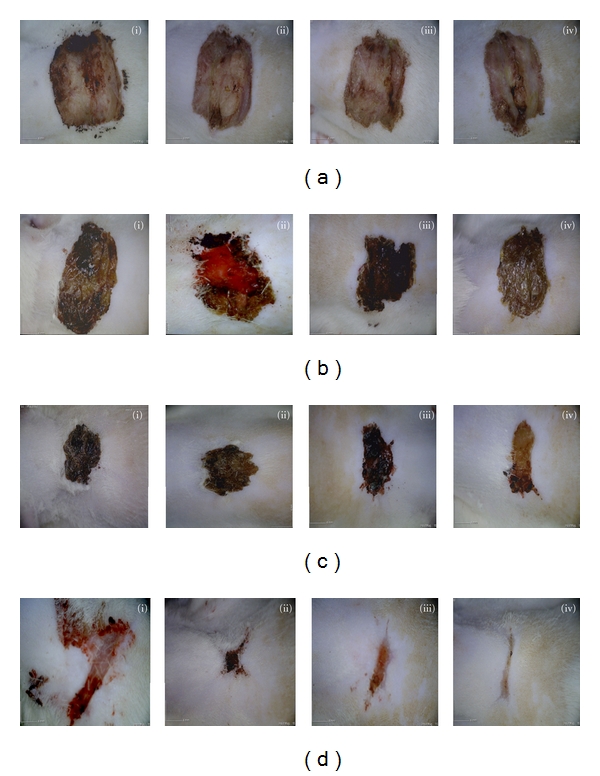
Excisional wound appearance after treatments: Results from (a) day 1 of treatments, (b) day 5 of treatments, (c) day 10 of treatments, and (d) day 15 of treatments; (i) untreated group, (ii) saline group, (iii) Intrasite Gel group, and (iv) Gelam honey group.

On day 1 of treatments (Figure 2(a), (i)–(iv)), there was enlargement of each wound but no macroscopic differences in all groups. Brownish thin scab was found on all wounds. On day 5 of treatments, bloody exudates were observed in saline group (Figure 2(b), (ii)), dry scab in untreated group (Figure 2(b), (i)) and Intrasite Group (Figure 2(b), (iii)), whilst moist scab in Gelam group (Figure 2(b), (iv)). Stiff, intact dark brown scabs were found in untreated, saline, and Intrasite groups (Figure 2(c), (i)–(iii)), while detachment of scab was found in Gelam group (Figure 2(c), (iv)) on day 10 of treatments. Fewer scars were found in Gelam group (Figure 2(d), (iv)) on day 15 of treatments, while wound healing process was still incomplete in untreated group (Figure 2(d), (i)).

**Figure 3:**
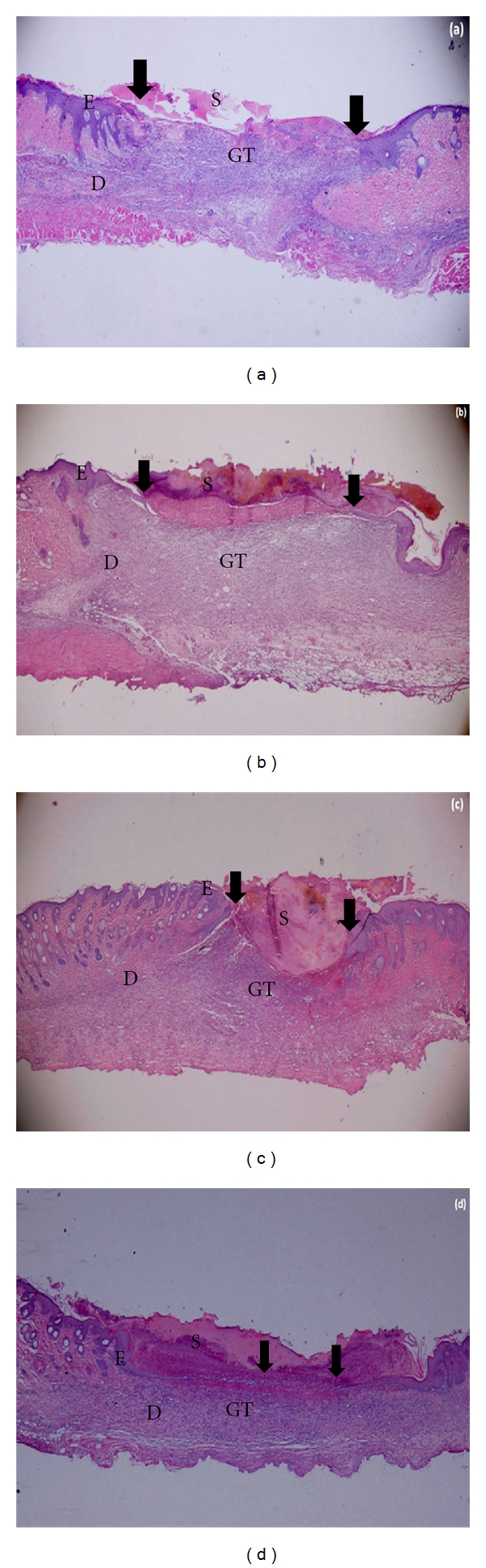
Photomicrographs of wound tissues at day 10 of treatments stained with H & E, 20x magnification: (a) Untreated Group, (b) Saline Group, (c) Intrasite Group, (d) Gelam Group. S—Scab; E—Epidermis; D—Dermis; GT-Granulation tissue. The arrow showed epithelialization.

The histological sections demonstrated the process of healing reflected by the macroscopical evaluation.  Figure 3 showed sections from day 10 of treatments. New epidermis formed in Gelam-honey-treated wounds was thinner and covered the entire wound area (Figure 3(d)). This provided protection to the wound from further injuries. Epithelial regeneration in Intrasite Gel was faster, but the scab on the surface was thick (Figure 3(c)). For nontreatment and saline-treated groups, the epithelialization was just at the early phase (Figures 3(a) and 3(b)). Epithelialization was showen in Figure 3 by the arrows.

### 3.3. Measurement of Wound Contraction (%)

**Figure 4:**
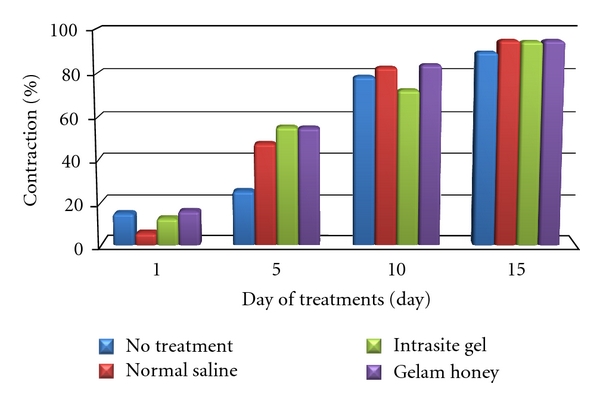
Contraction of wound area as percentage of original wound.

The results showed a trend of progressive contraction from day 5 onwards for both longitudinal and transverse wound contraction. Percentage of wound area contraction of each treatment was shown in Figure 4. The percentage of contraction for wound areas in all groups was significantly increased along the duration of wound healing. By day 10, the nontreatment group has “caught up” to the other groups regarding the healing (wound contraction percentage). It seemed like the relevant action occurred between day 5 and 10. Saline group had similar percentage of wound contraction as the Gelam group by day 10.

As expected, there was no significant difference (*P* < 0.05) between the groups on day 1 of treatments. Comparatively, by day 5 of treatments, the contraction of wound area for each was significantly increased with wound contraction in Intrasite group and Gelam group significantly (*P* < 0.05) greater compared to untreated group and saline group. Later in day 10 of treatments the contraction of wound in Gelam group was significantly greater compared to Intrasite group. However, by day 15 of treatments there was no statistically significant result in wound contraction among the groups.

## 4. Discussion

Simple and reproducible wound model is essential for wound research. Incisional and excisional wounds are the two main wound models in wound research which allowed the determination of the wound healing phases. Full thickness excisional wounds were used in this study to macroscopically and histologically evaluate the efficacy of topical application of Gelam honey (a Malaysian honey) in facilitating wound healing. The excisional wound was found to be more suitable for histological evaluation due to the broader morphological changes occurring during the process [13].

The results of this study showed that topical application of Gelam honey significantly accelerated the rate of wound healing compared to the saline treated group. This is similar to findings on the efficacy of topical application of honey in wound management as reported by many researchers [2, 5–7]. Acceleration of the healing rate might be due to the characteristics of honey (such as production of hydrogen peroxide and its nutritional, hydroscopic, antioxidant, and antibacterial properties) that provided wounds with suitable environment for promoting healing process [2, 14]. Published reports described that honey helped in clearing infection and protecting wound from being infected [15]. Since activities of honey depended on their nectar source [2, 16], thus, it seemed that the flora source of Gelam honey had positive effects in wound healing. The positive effects correlated wound healing with antibacterial properties of different types of honey in accelerating wound healing as had been tested in animal experiments [10, 17]. Beside Gelam honey, as expected, Intrasite Gel also accelerated the rate of healing in this study. Although both treatments accelerated wound healing, the application of Gelam honey was simple and inexpensive.

This study demonstrated that topical application of Gelam honey reduced the hard, intact dark brown scab in wound healing if compared to other treatment groups (Figure 2). Thus, Gelam-honey-treated wounds appeared to be clean and healthy looking in contrast to hard scabs observed on Intrasite-Gel-treated wounds. Comparatively, scabs on the honey-treated wounds were also easily detached and the scar was thinner. This could be attributed by the viscosity of honey which provided moist healing environment, promoting healing process by enhancing epithelialization and providing antibacterial barrier protection [2]. The results of this study are in agreement with the positive effects of the efficacy of Malaysian honey in wound healing as reported by previous animal studies [2, 10].

Wound contraction is an essential process in healing that lead to wound closure. Thus, visible appearances and measurements of wound contraction become reliable parameters in macroscopic evaluation for wound healing [13]. This study showed that Gelam honey significantly stimulated the contraction of wounds as seen from wound area (Figure 4). According to Aljady et al. [2] and Yusof et al. [15], honey accelerated wound healing by enhancing wound contractions. It stimulates wound contraction by providing energy needed for contractile activity. Besides that, it enhances the deposition of fibroblast and collagen which is the main factor for healing [2]. The greater the wound contraction, the lesser the scar deposition [18].

Histological evaluation showed significant epithelial regeneration appearance in Gelam-honey-treated wound. The epithelialization process was almost complete in Gelam honey treatment compared to other treatment groups (Figure 3(d)). Gelam honey might have the properties of enhancing the rate of healing of various phases, such as increased collagen formation and angiogenesis [13, 18]. Honey contains high levels of glycine, methione, arginine and proline which is essential in collagen formation and deposition [2, 19].

## 5. Conclusion

This study demonstrated the efficacy of Gelam honey in wound healing. Topical application of Gelam honey accelerated the rate of wound healing by increasing the wound contraction. The acceleration of wound healing was demonstrated by the healing rate and wound contraction through macroscopic and histological evaluation. Possibly, the relevant actions regarding the healing (wound contraction percentage) seemed to be occurring between days 5 and 10. Hence, Gelam honey is potentially useful in dressing wounds but further studies are needed to detail out the specific mechanisms of the honey.

## Figures and Tables

**Table 1:** Time required for wound healing in rats.

Type of dressing and grouping	Healing time (days) Mean ± S.E.M.
No treatment (untreated group)	16.67 ± 0.80^a^
Normal saline (saline group)	15.83 ± 0.40^a^
Intrasite Gel (Intrasite group)	13.00 ± 0.37^b^
Gelam honey (Gelam group)	13.17 ± 0.48^b^

All values were expressed as mean and standard error mean (S.E.M.); a value of *P* < 0.05 was considered significant. Means with different superscripts (a, b) were significantly different (*P* < 0.05).
